# Discordance of PD-L1 expression between primary and paired metastatic non-small cell lung cancer: a systematic review and meta-analysis

**DOI:** 10.3389/fimmu.2026.1942041

**Published:** 2026-10-20

**Authors:** Leichao Zhang, Yiyao Gao, Xuemei Wang, Yu Gu, Lijian Hou, Xueju Wang

**Affiliations:** 1 Department of Pathology, China-Japan Union Hospital of Jilin University, Changchun, Jilin, China; 2 Science Research Center, China-Japan Union Hospital of Jilin University, Changchun, Jilin, China

**Keywords:** biomarker, immunotherapy, non-small cell lung cancer, PD-L1, tumor heterogeneity

## Abstract

**Background:**

PD-L1 expression in non-small cell lung cancer (NSCLC) serves as a key predictive biomarker of response to immune checkpoint inhibitors; however, the concordance of PD-L1 expression between primary tumors and their corresponding metastatic lesions remains uncertain. We therefore performed a systematic review and meta-analysis to evaluate the variability of PD-L1 expression across paired primary and metastatic NSCLC sites.

**Methods:**

We systematically searched the Cochrane Library, Embase, PubMed, and Web of Science for eligible studies. Pooled discordance rates and patterns of mismatch were estimated using a random-effects model, and subgroup analyses were conducted according to PD-L1 cutoff, antibody clone, and metastatic site.

**Results:**

Fifteen studies fulfilled the inclusion criteria, encompassing 1,029 matched primary–metastatic sample pairs from 1,014 patients with NSCLC. The pooled analysis revealed an overall PD-L1 discordance rate of 21% between primary tumors and corresponding metastases (95% CI: 18%–25%). Notably, transitions from PD-L1-positive primary tumors to PD-L1-negative metastatic lesions occurred more frequently (26%, 95% CI: 22%–30%) than the reverse pattern, from negative primaries to positive metastases (18%, 95% CI: 12%–24%). Discordance of similar magnitude was observed across PD-L1 positivity thresholds and antibody clones and in both lymph node and brain metastases, although the site-specific estimates are exploratory.

**Discussion:**

In patients with NSCLC, PD-L1 expression frequently differs between primary and metastatic lesions, with a greater tendency for expression to decrease at metastatic sites. These findings indicate that PD-L1 results obtained from an archival primary tumor should be interpreted with caution when they are used to inform treatment of metastatic disease. Whether repeat testing of metastatic tissue improves patient selection or immunotherapy outcomes has not been demonstrated and requires prospective evaluation.

**Systematic review registration:**

https://www.crd.york.ac.uk/PROSPERO, identifier CRD42022336191.

## 1 Introduction

Lung cancer is the second most commonly diagnosed malignancy worldwide and remains the leading cause of cancer-related mortality. Despite substantial progress in early detection and treatment, the overall prognosis remains poor, with a five-year survival rate of approximately 20% (1). Moreover, the global incidence of lung cancer continues to rise across many regions. Non-small cell lung cancer (NSCLC) accounts for approximately 85% of all newly diagnosed lung cancer cases (2, 3). Among NSCLC subtypes, adenocarcinoma and squamous cell carcinoma are the most common histological types, each exhibiting distinct epidemiological, molecular, and biological characteristics. The high mortality rate of NSCLC is largely attributable to late-stage diagnosis, with most patients presenting with locally advanced or metastatic disease, which underscores the urgent need for improved systemic therapeutic strategies (4). In recent years, the therapeutic landscape of NSCLC has undergone a substantial transformation. The traditional reliance on chemotherapy has gradually given way to more personalized interventions, incorporating molecularly targeted therapies and immune-based treatments (5). In particular, immune checkpoint inhibitors (ICIs) targeting the PD-1/PD-L1 signaling axis have demonstrated marked therapeutic benefit in selected NSCLC patients (6, 7). By blocking the PD-1/PD-L1 interaction, ICIs restore and enhance the ability of the immune system to recognize and eliminate cancer cells, thereby producing durable clinical responses and improved survival outcomes (8). Consequently, ICIs have become a cornerstone of treatment for advanced NSCLC, used either as monotherapy or in combination with chemotherapy (9, 10). Nevertheless, the efficacy of ICIs varies considerably among patients, with response rates typically ranging from 20% to 50% (11). This variability is partly attributable to the dynamic and complex nature of PD-L1 expression within the tumor microenvironment (12). PD-L1 levels are influenced both by intrinsic factors, such as oncogenic pathways, and by extrinsic factors, including interferon-gamma released by tumor-infiltrating lymphocytes, which together result in considerable spatial and temporal heterogeneity. Furthermore, PD-L1 expression may be modulated by tumor mutational burden, microsatellite instability, and the presence of specific oncogenic driver mutations (13, 14). As a result, PD-L1 expression can vary substantially not only between primary tumors and their metastases but also among different metastatic sites (15, 16).

The variability of PD-L1 expression presents a major challenge to its use as a reliable predictor of ICI response in NSCLC. At present, immunohistochemistry (IHC) remains the standard technique for assessing PD-L1 in tumor specimens (17). However, because tissue acquisition is invasive, PD-L1 evaluation is often performed on small biopsy specimens or archived samples, which may not adequately capture the spatial heterogeneity of PD-L1 expression within the tumor (18). Furthermore, in patients with advanced NSCLC, metastatic lesions frequently constitute the sole source of tissue available for PD-L1 testing, as the primary tumor may have been surgically resected or may be inaccessible (19). Understanding the concordance of PD-L1 expression between primary tumors and metastatic sites is therefore essential for optimizing immunotherapy strategies in NSCLC. To address this knowledge gap, we conducted a systematic review and meta-analysis to quantify the extent of PD-L1 discordance between matched primary and metastatic NSCLC lesions and to explore the underlying mechanisms and clinical implications of such discordance. Notably, to our knowledge, at the time of our search the present study was the largest pooled analysis of paired primary and metastatic NSCLC samples, providing a comprehensive evaluation of PD-L1 heterogeneity in this disease setting.

This review begins with a synthesis of the current evidence on PD-L1 expression in primary and metastatic NSCLC, highlighting key findings from the existing literature. We then present the results of our systematic review and meta-analysis, focusing on PD-L1 prevalence and discordance between matched tumors. Factors influencing these differences—including tumor subtype, metastatic site, and testing methodology—are subsequently examined. Finally, we discuss the clinical implications of our findings and propose directions for future research. Our overall aim is to clarify the patterns of PD-L1 variability in NSCLC, thereby informing immunotherapy decision-making and supporting the advancement of personalized treatment strategies.

## 2 Materials and methods

### 2.1 Study design and systematic review protocol

This systematic review and meta-analysis of diagnostic concordance was designed and reported in accordance with the Preferred Reporting Items for Systematic Reviews and Meta-Analyses (PRISMA) statement. The review protocol was registered with PROSPERO (registration no. CRD42022336191; submitted on 29 May 2022 and registered on 10 June 2022). The analyses differ from the registered protocol in the following respects: the protocol specified a logistic-normal random-effects model, whereas inverse-variance random-effects models were used (Section 2.6); PD-L1 positivity rates were analyzed in addition to the registered outcome, the discordance rate; the planned subgroup analyses by cancer type and sample size, and the planned meta-regression, were not performed; and funnel plots with Egger’s test, the sensitivity analysis restricted to studies using the antibody clone of a regulatory-approved assay, and the trim-and-fill analysis were added *post hoc*. All included studies were published, peer-reviewed articles; no unpublished data, submitted manuscripts or personal communications were used.

### 2.2 Review question and eligibility framework (PICOS)

The review question was framed using the PICOS structure. Population: adult patients with histologically confirmed non-small cell lung cancer (NSCLC) for whom matched primary tumor and metastatic tissue samples were available. Index test (intervention): immunohistochemical assessment of PD-L1 expression in the metastatic lesion. Comparator: immunohistochemical assessment of PD-L1 expression in the paired primary tumor of the same patient, using the same antibody clone and positivity threshold. Outcomes: the pooled PD-L1 positivity rate at each site, the pooled discordance rate between paired samples, and the direction of any discordance (positive primary to negative metastasis, or negative primary to positive metastasis). Study designs: prospective cohort studies and retrospective analyses reporting paired, patient-level PD-L1 data.

### 2.3 Search strategy and data sources

A comprehensive literature search was performed across the Cochrane Library, Embase, Medline (via PubMed), and Web of Science. The search terms incorporated MeSH headings such as “lung neoplasms,” “non-small-cell lung carcinoma,” “metastasis,” “recurrence,” “programmed death 1 receptor,” and “B7-H1 antigen.” Truncation and proximity operators were used where appropriate, and the search covered English-language full-text articles published up to January 2022.

### 2.4 Eligibility criteria

Studies were eligible for inclusion if participants had histologically confirmed NSCLC. Only English-language, peer-reviewed articles providing complete methodological information were considered. Eligible studies were required to report comparative immunohistochemical PD-L1 expression data from matched primary tumors and metastatic sites, including either regional lymph nodes or distant metastases. Studies involving human subjects—both prospective cohorts and retrospective analyses—were considered, provided that sufficient data were available for quantitative synthesis. Case reports, editorials, clinical guidelines, opinion articles, narrative reviews, studies enrolling fewer than 11 subjects, and previously published meta-analyses were excluded. To minimize analytical variation within pairs, both members of each matched pair were required to have been assessed within the same study using the same antibody clone, the same staining platform and the same positivity threshold. Studies in which the primary tumor and the metastasis had been evaluated with different assays or different cutoffs were not eligible. Assays, platforms and thresholds nevertheless differed between studies, and this between-study variation was examined in the subgroup and sensitivity analyses described below.

During the initial study selection in February 2022, two investigators (LCZ and YYG) independently screened all retrieved records. Following an initial review to identify potentially relevant studies, both reviewers applied the predefined inclusion criteria independently and subsequently collaborated to finalize the list of eligible articles. Duplicate records were removed using the automatic detection function in EndNote 20, supplemented by manual verification. Any disagreements between reviewers were resolved through discussion, with adjudication by the senior researcher (XJW) when consensus could not be achieved.

### 2.5 Study selection and data extraction

For each included study, key information was extracted, including author names, year of publication, country of origin, study design (retrospective or prospective), and study setting (single-center or multicenter). Patient demographics and clinical characteristics extracted included cohort size, the number of matched primary–metastasis pairs, median age, sex distribution, and smoking history. Additional variables recorded included metastatic sites, tissue acquisition method (resection or biopsy), tumor histology, prior treatments, and details of PD-L1 evaluation, such as the IHC platform, antibody clone, positivity thresholds, and timing of metastasis (synchronous or metachronous). PD-L1 expression in tumor cells (TC) and tumor-infiltrating lymphocytes (TIL) was assessed separately for primary and metastatic tumors, and expression levels were stratified into <1%, 1–49%, or ≥50%. Positivity criteria were defined according to each study’s individual cutoffs, and the concordance rates of PD-L1 status between matched samples were recorded (see **Table 1**). Study quality was assessed using the QUADAS-2 tool (27), which evaluates four key domains—patient selection, index test methodology, reference standard accuracy, and consistency in the timing of sample analysis—each appraised for risk of bias.

**Table 1:** PD-L1 expression in the primary and paired metastasis and discordance status.

Author	Publish year	N	IHC system	Antibody used	Threshold for positivity	PD-L1 in TCs (primary lesion)			PD-L1 in ICs (primary lesion)			PD-L1 in TCs (primary lesion)		PD-L1 in TCs (metastasis)			PD-L1 in ICs (metastasis)			PD-L1 in TCs (metastasis)		Concordance in TCs (n [%])	Discordance in TCs (n [%])	Concordance in ICs (n [%])	Discordance in ICs (n [%])
						<1%	1-49%	≥50%	<1%	1-49%	≥50%	Positive	Negative	<1%	1-49%	≥50%	<1%	1-49%	≥50%	Positive	Negative				
Kim S (6)	2017	161	Ventana Benchmark XT	E1L3N	≥1% in tumor cells	117	24	20	NA	NA	NA	44	117	119	19	23	NA	NA	NA	42	119	129(80%)	32(20%)	NA	NA
Xu (13)	2019	76	DAKO Link48	22C3	≥1% in tumor cells	45	23	8	NA	NA	NA	31	45	45	21	10	NA	NA	NA	31	45	56(74%)	20(26%)	NA	NA
Wei (14)	2022	36(Group2)	Manual staining	E1L3N	≥1% in tumor cells	25	NA	NA	NA	NA	NA	11	25	25	NA	NA	NA	NA	NA	11	25	30(83%)	6(17%)	NA	NA
		57(Group3)	Manual staining	E1L3N	≥1% in tumor cells	41	NA	NA	NA	NA	NA	16	41	40	NA	NA	NA	NA	NA	17	40	50(88%)	7(12%)	NA	NA
Mansfield (15)	2016	73	Leica Bond RX	E1L3N	≥5% in tumor cells, ≥5% in immune cells	NA	NA	NA	NA	NA	NA	32	41	NA	NA	NA	NA	NA	NA	24	49	63(86%)	10(14%)	54(74%)	19(26%)
Takamori (16)	2017	21	DAKO EnVision	SP142	≥5% in tumor cells	NA	NA	NA	NA	NA	NA	8	13	NA	NA	NA	NA	NA	NA	7	14	16(76%)	5(24%)	NA	NA
Uruga (17)	2017	33(pN1)	Manual staining	E1L3N	≥1% in tumor cells	13	12	8	NA	NA	NA	20	13	16	10	7	NA	NA	NA	17	16	28(85%)	5(15%)	NA	NA
		32(pN2)	Manual staining	E1L3N	≥1% in tumor cells	13	15	4	NA	NA	NA	19	13	13	14	5	NA	NA	NA	19	13	20(62.5%)	12(37.5%)	NA	NA
Munari (18)	2018	75	Ventana Benchmark ULTRA	SP263	≥1% in tumor cells	42	21	12	NA	NA	NA	33	42	47	19	9	NA	NA	NA	28	47	66(88%)	9(12%)	NA	NA
Zhou (19)	2018	25	Shuwen Biotech Co	6E8	≥5% in tumor cells, ≥5% in immune cells	NA	NA	2	NA	NA	6	17	8	NA	NA	6	NA	NA	11	16	9	18(72%)	7(28%)	15(60%)	10(40%)
Téglási (20)	2019	61	Leica Bond-Max	SP142	≥1% in tumor cells, ≥1% in immune cells	39	18	4	34	NA	NA	22	39	40	10	11	38	NA	NA	21	40	NA	NA	NA	NA
Batur (21)	2020	24	DAKO Link 48	22C3	≥1% in tumor cells, ≥1% in immune cells	6	9	9	6	13	5	18	6	7	9	8	6	16	2	17	7	19(79%)	5(21%)	18(75%)	6(25%)
Luo (22)	2020	30(untreated)	Dako PT100	22C3	≥1% in tumor cells	15	9	6	NA	NA	NA	15	15	14	10	6	NA	NA	NA	16	14	23(77%)	7(23%)	NA	NA
		80(treated)	Dako PT100	22C3	≥1% in tumor cells	40	22	18	NA	NA	NA	40	40	34	32	14	NA	NA	NA	46	34	58(72.5%)	22(27.5%)	NA	NA
Hwang (23)	2021	27	Dako Link48	22C3	≥1% in tumor cells	11	5	11	NA	NA	NA	16	11	12	7	8	NA	NA	NA	15	12	20(74%)	7(26%)	NA	NA
Shen (24)	2021	49	Dako Link48	22C3	≥1% in tumor cells	22	18	9	NA	NA	NA	27	22	29	8	12	NA	NA	NA	20	29	38(78%)	11(22%)	NA	NA
Inoue (25)	2016	132	Manual staining	E1L3N	almost equivalent to ≥5% in tumor cells	NA	NA	NA	NA	NA	NA	54	78	NA	NA	NA	NA	NA	NA	71	61	85(64%)	47(36%)	NA	NA
Kim H (26)	2017	37	Ventana Bench Mark XT	SP142	≥5% in tumor cells	NA	NA	4	NA	NA	NA	7	30	NA	NA	2	NA	NA	NA	8	29	30(81%)	7(19%)	NA	NA

N, number; NA, not available; PD-L1, programmed death-ligand 1; TCs, tumor cells; ICs, immune cells.

### 2.6 Data analysis

The primary outcome was the pooled PD-L1 positivity rate in both primary and metastatic tumor sites, considering expression in tumor cells and immune cells. As a secondary objective, the rate of PD-L1 discordance between matched primary and metastatic samples was evaluated, accounting for differences in staining of either cell type. The analysis also examined the directionality of discordance, namely whether PD-L1 expression was more frequently elevated in primary tumors or in metastases.

Study-level positivity and discordance rates were calculated with exact (Clopper–Pearson) 95% confidence intervals and pooled by inverse-variance random-effects meta-analysis, with the between-study variance (τ^2^) estimated by restricted maximum likelihood. Positivity rates were pooled after log transformation and discordance rates as untransformed proportions; common-effect estimates are presented alongside for comparison. When substantial heterogeneity was detected, subgroup and sensitivity analyses were performed to identify potential contributing factors. Stratified analyses were carried out according to antibody clone, established positivity cutoffs, and metastatic site. An additional *post hoc* sensitivity analysis was performed in which the pooled discordance rate was re-estimated after restricting the dataset to studies that used the antibody clone of a regulatory-approved PD-L1 assay (22C3, SP263 or SP142), thereby excluding laboratory-developed tests based on the E1L3N and 6E8 clones; staining platforms were not uniform within this subset. Heterogeneity was assessed using the Q statistic and the I^2^ index (28), with values exceeding 50% indicating considerable variability. Differences between subgroups were evaluated using chi-square tests. Potential publication bias was investigated through funnel plots and Egger’s regression test, applied on the scale used for pooling to outcomes with at least ten datasets; where Egger’s test was significant, the trim-and-fill method was applied as a *post hoc* sensitivity analysis. Statistical significance was set at P < 0.05. All analyses were performed using Review Manager 5.4 and R (version 4.6.1) with the “meta” package (version 8.5-0).

## 3 Results

### 3.1 Study selection, study characteristics and assessment of risk of bias

Fifteen original studies were included in the meta-analysis (6, 13–26), encompassing 1,029 matched pairs of primary and metastatic NSCLC tissue samples from 1,014 patients (**Figure 1**). These retrospective studies, published between 2016 and 2022, comprised ten single-center and five multicenter investigations. Study characteristics are summarized in **Supplementary Table 1**, while PD-L1 expression data and discordance rates are detailed in **Table 1**. Reporting of patient demographics and treatment history varied across studies. Among the 666 patients for whom sex was reported, 65.3% were male, and median ages ranged from 57 to 63 years. Current or former smokers accounted for 56.9% of the cohort. Adenocarcinoma was the predominant histological subtype (81.7%), followed by squamous cell carcinoma (16.2%) and other variants (2.1%). The most frequent metastatic sites were regional lymph nodes (55.4%) and the brain (19.7%). The timing of sampling was reported in thirteen of the fifteen studies. Paired samples were obtained synchronously in five studies and metachronously in four; the remaining four included metachronous pairs in only part of the cohort, and in one of these the synchronous and metachronous subsets were enumerated separately. Treatment history was reported in eleven studies: some or all patients had received chemotherapy, targeted therapy, radiotherapy, microwave ablation or corticosteroids in seven, and no patient had been treated in four. The source studies rarely specified whether treatment preceded both samples or was given between them. One study reported treated and untreated cohorts separately, and these are analyzed as separate datasets throughout. Full details for each study are given in **Supplementary Table 1**. PD-L1 expression was predominantly assessed using the Dako platform, with the 22C3 and E1L3N antibody clones most commonly applied; the 6E8, SP263, and SP142 clones were used in a small number of studies. Study quality was evaluated according to the QUADAS-2 criteria (**Figure 2A**). Considerable heterogeneity was observed across studies. Funnel plots and Egger’s test showed little evidence of small-study effects for most outcomes (**Figure 2B**). The exceptions were PD-L1 positivity in metastases across all datasets (P = 0.030), for which less precise studies tended to report lower rates, and the rate of conversion from PD-L1-negative primary tumors to PD-L1-positive metastases (P = 0.004), for which less precise studies tended to report higher rates (random-effects estimate 18% versus common-effect estimate 12%). Trim-and-fill imputed six studies for metastatic positivity, two of which corresponded to proportions above 100% and the remaining four to proportions higher than in any observed study, and eight studies for negative-to-positive conversion, all of which corresponded to negative proportions; because such values are impossible, no interpretable adjusted estimate could be derived for either outcome. Because the standard error of a proportion depends on the proportion itself, imprecise estimates cluster among low proportions on the log scale and, for proportions below 50%, among high proportions on the untransformed scale, so asymmetry of this kind is expected without publication bias (29).

**Figure 1:**
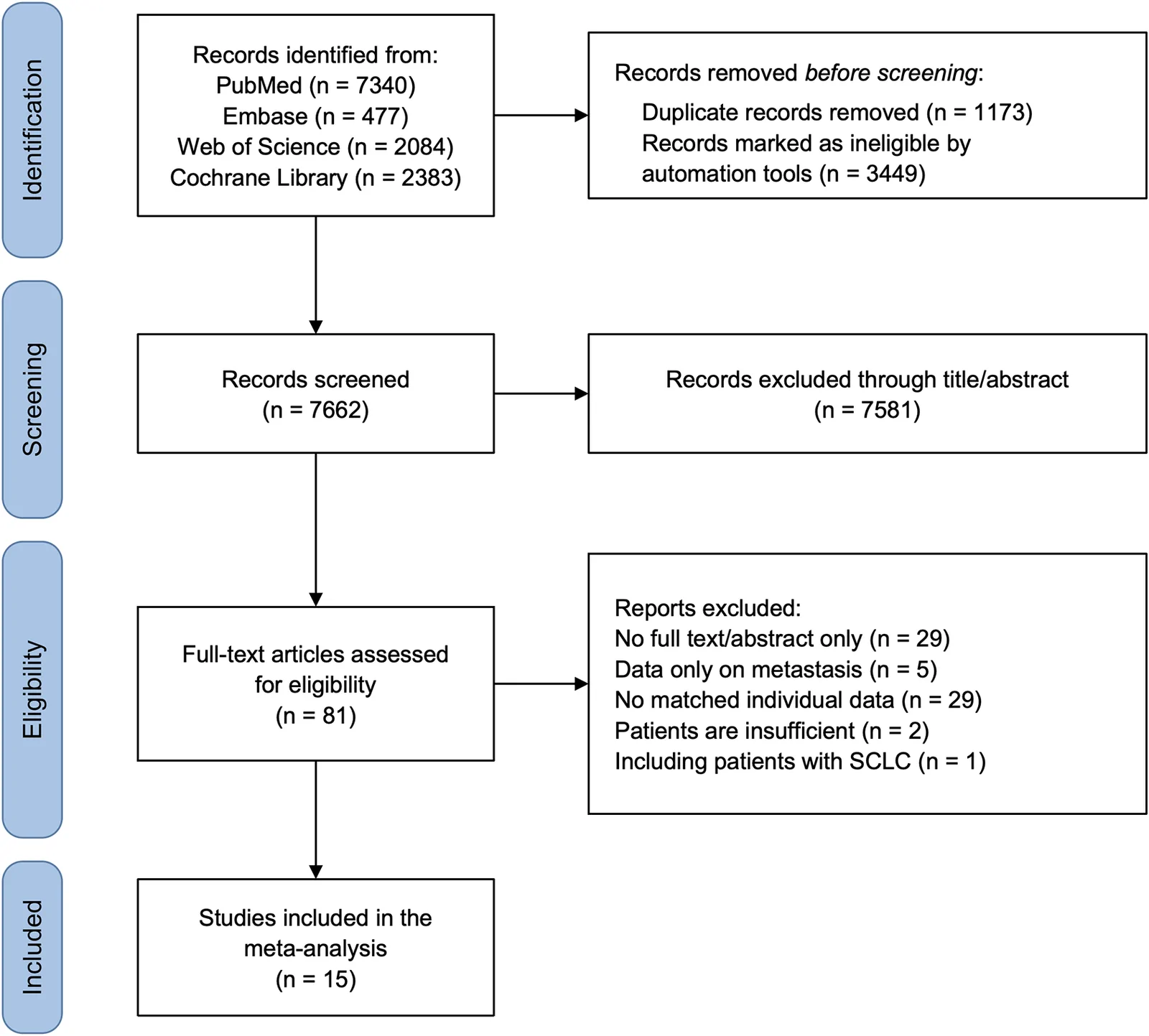
Flow diagram illustrating the search strategy and study selection process.

**Figure 2:**
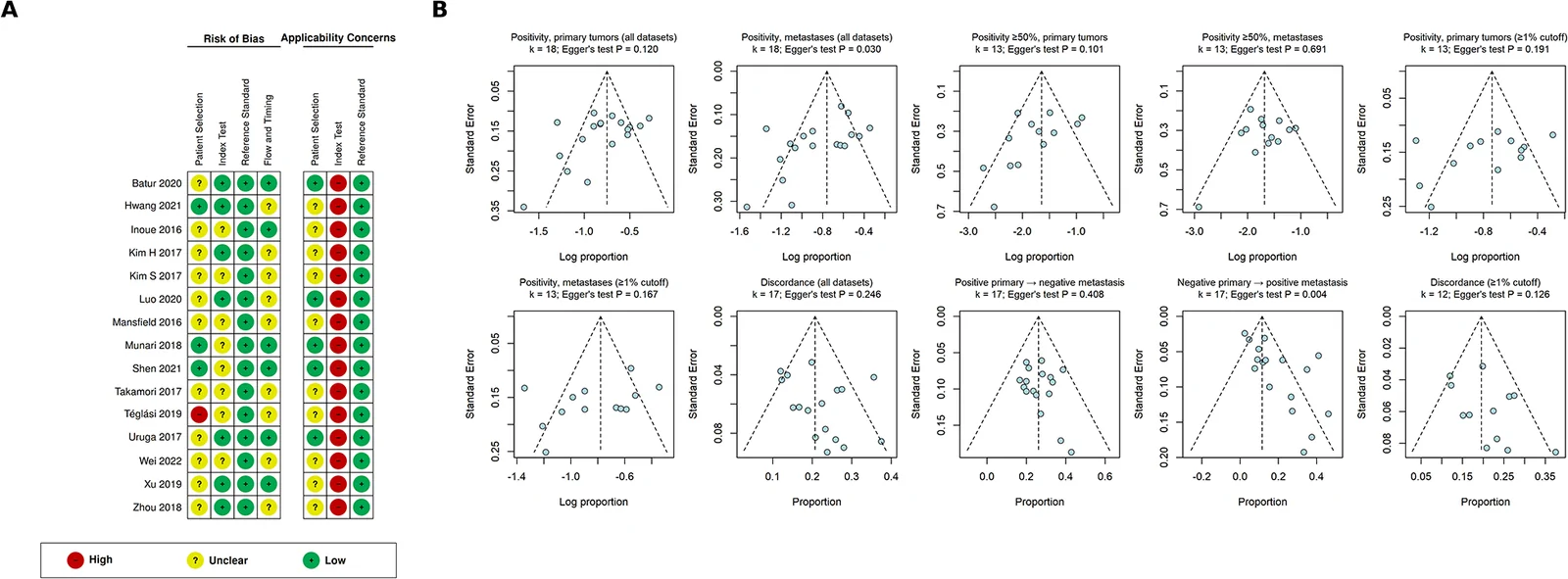
Assessment of study quality and publication bias. **(A)** Quality evaluation of included studies; **(B)** Funnel plots with Egger’s regression test for outcomes with at least ten datasets; positivity outcomes are shown on the log scale and discordance outcomes as untransformed proportions.

### 3.2 PD-L1 expression in paired primary tumor and metastasis

The overall PD-L1 positivity rate in tumor cells was 45% in primary NSCLC tumors (95% CI: 39–52%; 15 studies, 18 datasets; n = 1,029; I^2^ = 77%) (**Figure 3A**), compared with 43% in metastatic lesions (95% CI: 37–50%; same studies and datasets; n = 1,029; I^2^ = 77%) (**Figure 3B**). Using a ≥50% cutoff, PD-L1 positivity was slightly higher in metastases at 19% (95% CI: 15–23%; 11 studies, 13 datasets; n = 710; I^2^ = 30%) (**Figure 3D**) than in primary tumors at 18% (95% CI: 13–24%; 11 studies, 13 datasets; n = 710; I^2^ = 66%) (**Figure 3C**).

**Figure 3:**
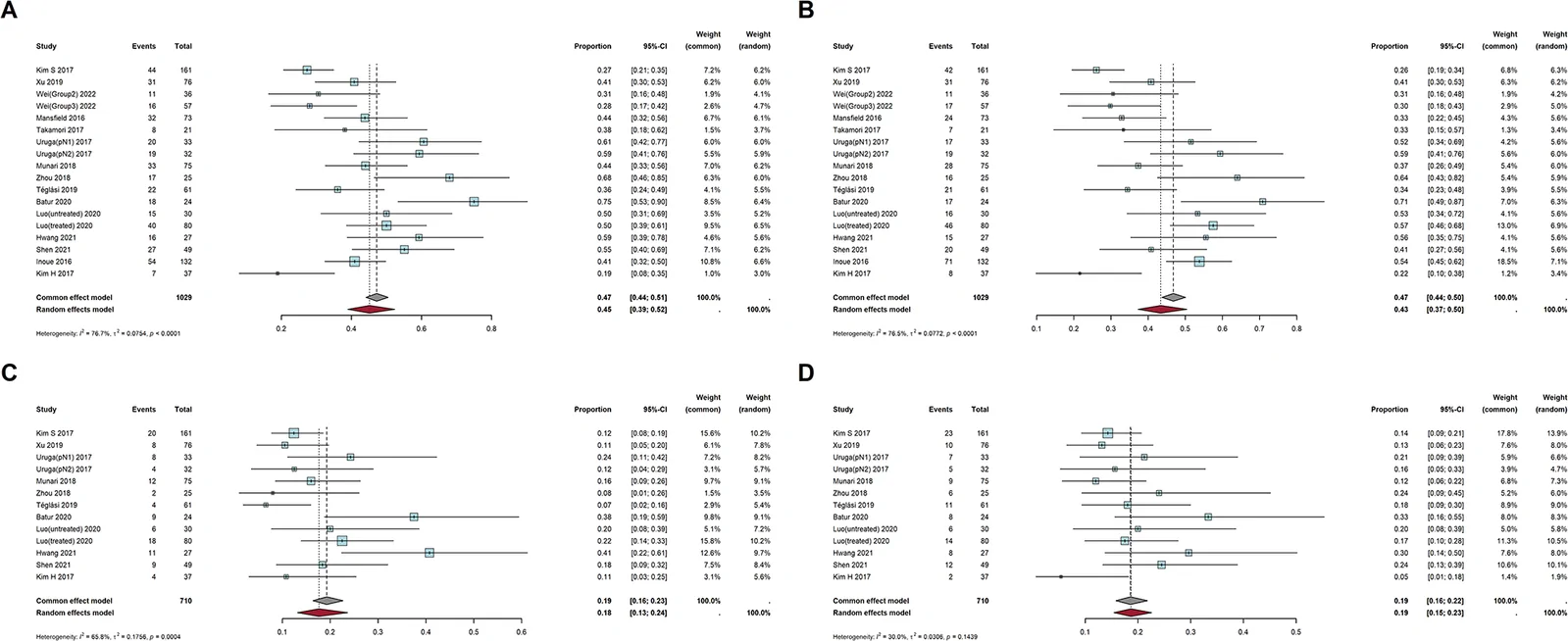
Pooled PD-L1 positivity rates. **(A)** PD-L1 positivity in tumor cells of primary tumors; **(B)** PD-L1 positivity in tumor cells of metastatic lesions; **(C)** PD-L1 positivity in primary tumors at the ≥50% cutoff; **(D)** PD-L1 positivity in metastases at the ≥50% cutoff.

### 3.3 Discordance of PD-L1 expression between paired primary tumor and metastasis

Discrepancies in PD-L1 expression between matched primary tumors and metastases were analyzed, with detailed results presented in **Supplementary Table 2**. The overall discordance rate in tumor cells was 21% (95% CI: 18–25%), based on 14 studies encompassing 17 datasets (n = 968; I^2^ = 54%) (**Figure 4**). Notably, loss of PD-L1 expression from positive primary tumors to negative metastases was more common than the reverse pattern. The pooled discordance rate for PD-L1-positive primaries shifting to PD-L1-negative metastases was 26% (95% CI: 22–30%; 14 studies, 17 datasets; n = 408; I^2^ = 0%), whereas the conversion from PD-L1-negative primaries to PD-L1-positive metastases occurred at a lower rate of 18% (95% CI: 12–24%; 14 studies, 17 datasets; n = 560; I^2^ = 78%).

**Figure 4:**
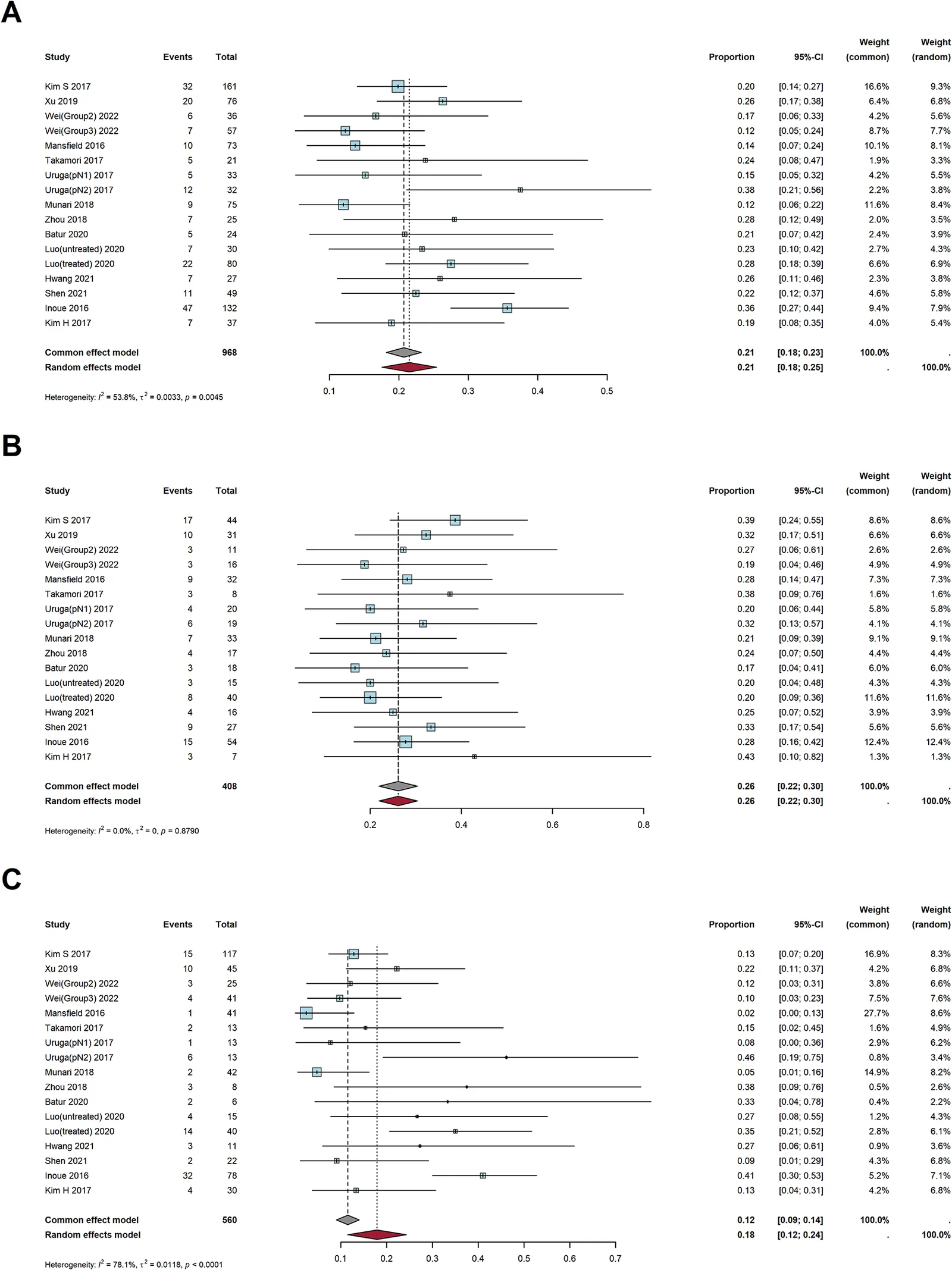
PD-L1 expression discordance between matched primary tumors and metastases. **(A)** Overall discordance rate in tumor cells; **(B)** Discordance from PD-L1-positive primary tumors to PD-L1-negative metastases; **(C)** Discordance from PD-L1-negative primary tumors to PD-L1-positive metastases.

### 3.4 Positivity and discordance rate of PD-L1 expression in paired primary tumor and metastasis in subgroups of interest

Subgroup analyses were performed according to PD-L1 cutoff values, antibody clones, and metastatic sites (**Supplementary Table 3**). The cutoff threshold notably influenced PD-L1 positivity rates in tumor cells. At the ≥1% cutoff, the pooled positivity was 46% in primary tumors (95% CI: 39–55%; 10 studies, 13 datasets; n = 741; I^2^ = 78%) (**Figure 5A**) and 44% in metastases (95% CI: 37–52%; same studies and datasets; n = 741; I^2^ = 77%) (**Figure 5B**). At the 5% cutoff, the positivity rates were slightly lower—42% in primary tumors (95% CI: 30–59%; 5 studies; n = 288; I^2^ = 76%) and 41% in metastases (95% CI: 28–59%; 5 studies; n = 288; I^2^ = 78%) (**Figures 5A, B**). Overall, PD-L1 positivity was marginally higher in primary tumors than in metastases. The discordance rate between paired samples was 20% at the 1% cutoff (95% CI: 16–24%; 9 studies, 12 datasets; n = 680; I^2^ = 37%) and increased to 24% at the 5% cutoff (95% CI: 15–33%; 5 studies; n = 288; I^2^ = 74%) (**Figure 5C**). Of note, heterogeneity was substantial at the 5% cutoff but only moderate at the 1% threshold.

**Figure 5:**
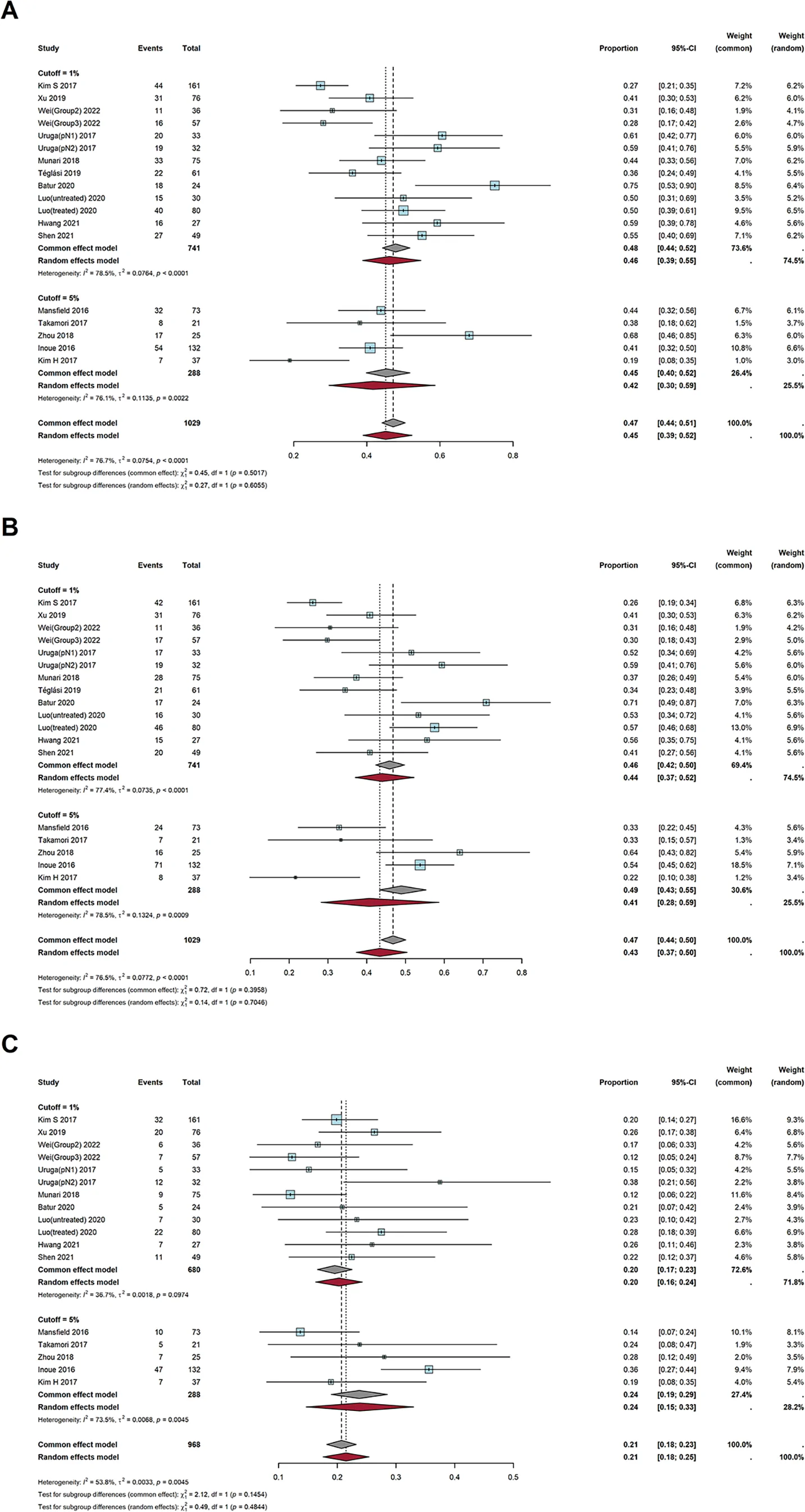
Subgroup analyses of PD-L1 positivity and discordance in paired tumors. **(A)** PD-L1 positivity in primary tumors at the ≥1% and ≥5% cutoffs; **(B)** PD-L1 positivity in metastases at the ≥1% and ≥5% cutoffs; **(C)** Discordance rates between paired tumors at these cutoff levels.

When primary tumors and metastases were compared using the E1L3N antibody, the pooled PD-L1 positivity rate was 40% in primary tumors (95% CI 31–52%, 5 studies, 7 datasets, n = 524, I^2^ = 79%) and 39% in metastases (95% CI 30–51%, same studies and datasets, I^2^ = 83%) (**Figures 6B, C**). For the 22C3 clone, positivity rates were higher—54% in primary tumors (95% CI 46–65%, 5 studies, 6 datasets, n = 286, I^2^ = 61%) and 53% in metastases (95% CI 44–63%, same studies and datasets, I^2^ = 57%). With the SP142 clone, the rates were lower: 32% in primary tumors (95% CI 23–45%, 3 studies, n = 119, I^2^ = 38%) and 31% in metastases (95% CI 24–41%, same studies, I^2^ = 0%). The elevated positivity observed with 22C3 suggests that this clone may partly explain the variability across studies, whereas SP142 likely contributes to the observed heterogeneity. The discordance in PD-L1 expression detected using 22C3 was 25% (95% CI 20–30%, 5 studies, 6 datasets, n = 286, I^2^ = 0%), which was higher than the 21% observed with E1L3N (95% CI 14–28%, 5 studies, 7 datasets, n = 524, I^2^ = 76%) (**Figure 6A**). Owing to limited data, other antibody clones were not analyzed in detail. In the sensitivity analysis restricted to studies using the antibody clone of a regulatory-approved assay (22C3, SP263 or SP142; 8 studies, 9 datasets; n = 419), the pooled discordance rate was 22% (95% CI: 17–26%; I^2^ = 13%), compared with 21% (95% CI: 18–25%; I^2^ = 54%) in the full dataset.

**Figure 6:**
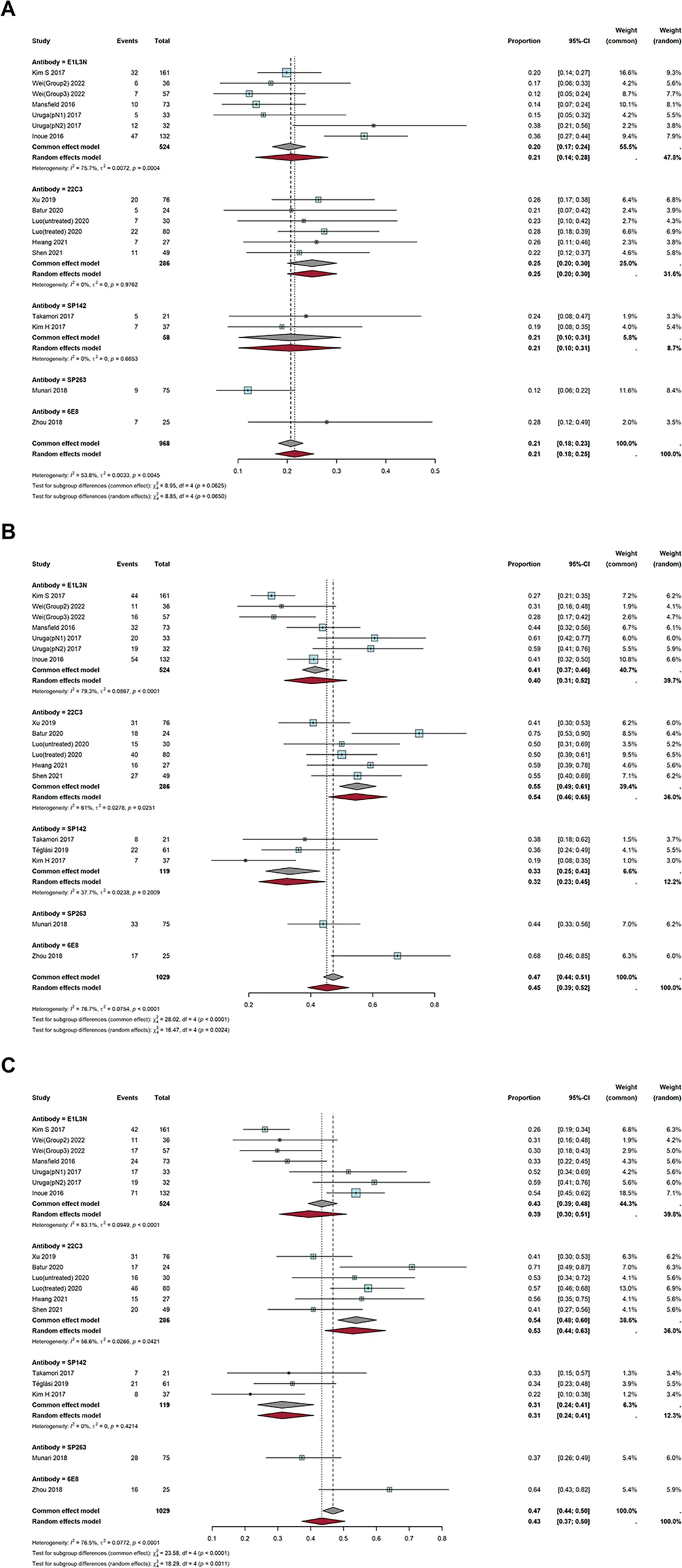
PD-L1 positivity and discordance stratified by antibody clone. **(A)** Discordance rates between paired tumors by clone; **(B)** Positivity rates in primary tumors by clone; **(C)** Positivity rates in metastases by clone.

When stratified by metastatic site, the pooled PD-L1 positivity rates in tumor cells exhibited the following patterns. Overall, primary tumors had a positivity rate of 50% (95% CI: 42–60%; 9 studies, 10 datasets; n = 541; I^2^ = 74%) (**Supplementary Figure 1A**), whereas metastases showed a rate of 47% (95% CI: 40–56%; same studies and datasets; I^2^ = 70%) (**Supplementary Figure 1B**). For regional lymph node metastases, the positivity was 48% in primary tumors (95% CI: 40–58%; 5 studies, 6 datasets; n = 358; I^2^ = 61%) and 47% in lymph nodes (95% CI: 40–56%; same studies and datasets; I^2^ = 48%) (**Supplementary Figures 1A, B**). Brain metastases exhibited positivity rates of 54% in primaries (95% CI: 38–76%; 4 studies; n = 183; I^2^ = 84%) and 48% in brain lesions (95% CI: 32–71%; same studies; I^2^ = 85%) (**Supplementary Figures 1A, B**). Considerable heterogeneity was present across these comparisons (I^2^ between 48% and 85%), and these site-stratified estimates should be regarded as exploratory. With regard to discordance, the pooled rate was 26% (95% CI: 18–33%; 5 studies, 6 datasets; n = 358; I^2^ = 61%) in regional lymph node metastases and 18% (95% CI: 10–26%; 3 studies; n = 122; I^2^ = 16%) in brain metastases (**Supplementary Figure 1C**). The difference between sites was not statistically significant (random-effects test for subgroup differences, P = 0.18), and, like the site-specific positivity estimates, these results should be regarded as exploratory.

## 4 Discussion

In the present study, a total of 15 studies were included, comprising 1,029 paired primary tumors and metastases from 1,014 patients with NSCLC. The overall discordance rate of PD-L1 expression between primary tumors and metastases was 21% (95% CI: 18–25%). Discordance was more frequently observed from PD-L1-positive primary tumors to PD-L1-negative metastases (26%, 95% CI: 22–30%) than from negative to positive (18%, 95% CI: 12–24%). Discordance of similar magnitude was observed across PD-L1 positivity thresholds and antibody clones and in both lymph node and brain metastases, although the site-specific analyses are exploratory.

Most lung cancer-related deaths occur because patients are diagnosed only after the disease has metastasized, underscoring the urgent need for improved systemic therapies to enhance long-term survival. Over the past two decades, the management of lung cancer has progressively shifted toward personalized therapeutic approaches (2). In 2015, a landmark milestone was achieved in advanced NSCLC management when the U.S. Food and Drug Administration (FDA) granted approval to the immune checkpoint inhibitor nivolumab for patients experiencing disease progression during or after platinum-based chemotherapy (30). This approval marked the beginning of a transformative era in lung cancer therapy. Since then, immune checkpoint inhibitors have demonstrated the potential to improve survival outcomes in selected patient populations (31). In 2019, the U.S. FDA expanded the indication for pembrolizumab to include first-line therapy for patients with stage III or metastatic NSCLC exhibiting a PD-L1 Tumor Proportion Score (TPS) of 1% or higher. The most commonly applied PD-L1 expression cutoffs in clinical research are 1%, 5%, and 50%, all of which were similarly adopted in our meta-analysis to assess PD-L1 positivity and discordance rates.

To our knowledge, at the time of our search our meta-analysis was the largest pooled compilation of studies and cases on this question, providing detailed insights into PD-L1 positivity in matched primary and metastatic NSCLC samples, the discordance between paired tumors, and the directionality of this discordance. Our results indicate that PD-L1 expression was generally lower at metastatic sites than in primary tumors. This pattern was consistent across various PD-L1 cutoffs (1% and 5%), antibody clones (E1L3N, 22C3, SP142), and, in exploratory analyses, metastatic sites (lymph nodes and brain), with the exception of the 50% cutoff. These findings are consistent with previous research demonstrating similar PD-L1 expression differences between primary and metastatic tumors in other malignancies, including breast cancer (12). Collectively, these observations highlight the spatial and temporal variability of PD-L1 expression. We further observed that discordance between matched primary and metastatic NSCLC tumors occurs bidirectionally: 26% of cases showed PD-L1-positive primary tumors paired with PD-L1-negative metastases, whereas 18% exhibited the opposite pattern. This reciprocal discordance mirrors observations reported in primary and metastatic breast cancer (12), reflecting the fluctuating and adaptable nature of PD-L1 expression. Such discordance may arise from differences in the tumor microenvironment, levels of immune cell infiltration, and the influence of prior therapies such as chemotherapy or radiation. In addition, factors such as genetic alterations, histological tumor subtypes, and the abundance of CD8+ tumor-infiltrating lymphocytes (TILs) may contribute to the observed variability in PD-L1 expression. For example, previous studies have shown that smoking status (32), prior radiotherapy (32), specific mutations (e.g., EGFR, KRAS) (33), and specimen type (resected, biopsy, or transbronchial needle aspiration) (24) can influence PD-L1 expression levels. Variations in PD-L1 detection assays, antibodies and scoring conventions (34–36) may further contribute to the observed heterogeneity. Consistent with this, restricting the analysis to studies using the antibody clones of regulatory-approved assays left the pooled discordance rate essentially unchanged (22% versus 21%) while between-study heterogeneity was lower (I^2^ = 13% versus 54%), which suggests that the laboratory-developed tests contributed to the heterogeneity of the full analysis rather than to the magnitude of discordance itself. Scoring convention warrants particular comment. Every study included here quantified staining in tumor cells, corresponding to the tumor proportion score (TPS), and several additionally recorded immune-cell staining separately. None reported the combined positive score (CPS), in which PD-L1-positive tumor cells, lymphocytes and macrophages are counted together and expressed relative to the number of viable tumor cells. In NSCLC, the companion diagnostic assays approved by the US Food and Drug Administration score PD-L1 on tumor cells—as the TPS for 22C3 and as the percentage of PD-L1-positive tumor cells for 28-8 and SP263—with SP142 additionally allowing an immune-cell criterion, whereas CPS underpins approvals in several other tumor types, including head and neck, gastroesophageal, cervical and triple-negative breast cancer. Because CPS incorporates the immune compartment, and because immune-cell PD-L1 expression appears to be more labile between paired lesions than tumor-cell expression (15, 20)—in the three included studies that reported both, concordance was lower for immune cells than for tumor cells (**Table 1**)—discordance measured by CPS need not match the tumor-cell discordance reported here. How CPS behaves across paired primary and metastatic NSCLC lesions has not been established and is a worthwhile subject for future work.

### 4.1 Limitations

Although comprehensive, our meta-analysis has several limitations. All included studies were retrospective in design, which may introduce bias. Some analyses involved relatively small patient numbers, potentially obscuring true effects. Moreover, the design of the included studies does not permit spatial heterogeneity to be separated from temporal change. A difference between a primary tumor and its paired metastasis may reflect genuine anatomical heterogeneity, but it may equally reflect the interval between the two biopsies, treatment given during that interval, or differences in specimen type between the two samples. Reporting of these variables was inconsistent across studies, and the intervals themselves were rarely quantified, so we were unable to stratify the pooled discordance estimate by timing or by interval therapy. The observed discordance should therefore be read as the combined effect of spatial and temporal variation rather than as a measure of spatial heterogeneity alone. Although publication bias was assessed with funnel plots and Egger's test, it cannot be fully excluded. The analysis relied on study-level rather than individual patient data, which may have contributed to the observed variability. The evidence remains mixed regarding whether metastatic PD-L1 status independently predicts immunotherapy outcomes; some studies suggest added prognostic value, whereas others do not. Notably, no prospective studies have directly linked metastatic PD-L1 discordance to clinical outcomes such as survival or response rates, highlighting a critical gap in the current literature. Relying solely on primary tumor PD-L1 status to guide treatment decisions is therefore problematic, especially given that metastatic samples often differ and are frequently the only tissue available after resection. This suggests that PD-L1 status determined on an archival primary tumor should be interpreted with caution in the metastatic setting. It does not, however, establish that testing the metastatic lesion yields better treatment decisions, since no study included here linked discordance to response or survival. In addition, our subgroup analyses primarily covered common metastatic sites (lymph nodes and brain), with limited data available for less frequent sites such as liver and bone. This limitation arises from the small number of studies that have examined PD-L1 expression at these locations. Organ-specific characteristics, such as the blood–brain barrier and differences in the immune microenvironment, may influence PD-L1 expression patterns. For example, the blood–brain barrier may restrict immune cell infiltration, leading to unique PD-L1 profiles in brain metastases compared with those at other metastatic sites (37). Similarly, the immune landscape in bone metastases may differ substantially from that in soft tissue or lymph node metastases. Such organ-specific differences suggest that the overall discordance rate reported in our meta-analysis may not be fully representative of all metastatic locations. Future studies should therefore aim to assess PD-L1 expression across a broader range of metastatic sites in order to capture more comprehensively the full extent of PD-L1 heterogeneity in NSCLC.

## 5 Conclusions

This meta-analysis evaluates PD-L1 expression and its variability between primary and metastatic NSCLC lesions and shows that discordance between paired lesions is common and occurs in both directions. These findings support standardized PD-L1 testing and careful interpretation of PD-L1 results obtained from archival primary tumors. Repeat assessment of PD-L1 may be informative where metastatic tissue is already available, for example from a clinically indicated biopsy or from comprehensive molecular profiling. Prospective evidence is nevertheless required before retesting can be expected to improve immunotherapy outcomes or be recommended as routine practice. Future research should focus on prospective studies linking metastatic PD-L1 discordance to clinical outcomes and on investigating the mechanisms underlying PD-L1 heterogeneity, including tumor microenvironment and organ-specific factors, to refine immunotherapy strategies in NSCLC.

## Data Availability

The original contributions presented in the study are included in the article/**Supplementary Material**. Further inquiries can be directed to the corresponding author.
