# Supplementary material for: Discordance of PD-L1 expression between primary and paired metastatic non-small cell lung cancer: a systematic review and meta-analysis
Source: Front Immunol. 2026 Oct 20;17:1942041. doi: 10.3389/fimmu.2026.1942041 (PMC13728784; doi:10.3389/fimmu.2026.1942041)
Supplement: Supplementary file 1 [file DataSheet1.docx]

Supplementary Material

# 1 Supplementary Figures and Tables

## 1.1 Supplementary Figures

**Supplementary Figure 1.** PD-L1 positivity and discordance by metastatic site. (A) Positivity rates in primary tumors across sites; (B) Positivity rates in metastases across sites; (C) Discordance rates between matched tumors by site.

Supplementary Tables

Supplementary Table 1 Characteristics of studies included in the meta-analysis.

| Author | Publish  Year | Country | Institution | The primary and  paired metastasis  N | Median age  [range] | M/F | Smoking status  (Ex‐)Smoker/Never/Unknown | Histology subtype  SCC/ADC/NOS | Tissue for PD-L1 analysis  resection/Biopsy  primary metastatic | Metastatic sites (N) | History of treatments (N) | Metastasis  Timing(N) |
| --- | --- | --- | --- | --- | --- | --- | --- | --- | --- | --- | --- | --- |
| Kim S | 2017 | Korea | Single-center | 161  (from 146 patients) | 63[27-80] | 81/65 | 61/67/18 | 0/146/0 | 146/0 146/15 | Mostly regional Lymph nodes (134) | neoadjuvant chemotherapy (15) | Metachronous  (34)  Synchronous (127) |
| Xu | 2019 | China | Single-center | 76 | 57.6[38-76] | 57/19 | 33/43/0 | 39/37/0 | 76/0 76/0 | Regional lymph nodes (76) | Preoperative chemotherapy (21) | Synchronous |
| Wei | 2022 | China | Multi-center | 36(Group2) | NR | 29/7 | 21/15/0 | NR/17/NR | 36/0 36/0 | Regional lymph nodes (36) | Some Chemotherapy, targeted therapy or MWA | NR |
|  |  |  |  | 57(Group3) | NR | 34/23 | 24/33/0 | NR/39/NR | 57/0 NR | Distant metastases (23) recurrent tumors (34) | Some Chemotherapy, targeted therapy or MWA | NR |
| Mansfield | 2016 | USA | Single-center | 73 | 61[54-69] | 40/33 | 58/15/0 | 17/54/2 | 63/10 70/3 | Brain (73) | NR | Metachronous |
| Takamori | 2017 | Japan | Single-center | 21 | 63[29-79] | 17/4 | 18/3/0 | 4/15/2 | 14/7 14/7 | Brain (16); adrenal gland (3); spleen (1); jejunum (1) | Chemotherapy for the primary tumor (5), radiotherapy for metastatic lesions (2). | Metachronous  (9) |
| Uruga | 2017 | USA | Single-center | 33(pN1) | NR | NR | NR | 0/33/0 | 33/0 33/0 | Regional lymph nodes (33) | None | Synchronous |
|  |  |  |  | 32(pN2) | NR | NR | NR | 0/32/0 | 32/0 32/0 | Regional lymph nodes (32) | None | Synchronous |
| Munari | 2018 | Italy | Single-center | 75 | NR | NR | NR | NR | All resection, but one biopsy | Regional lymph nodes (71), distant sites (4) | None | Metachronous  (4) |
| Zhou | 2018 | China | Multi-center | 25 | 57[26-78] | 18/7 | 12/13/0 | 3/20/2 | 23/2 25/0 | Brain (25) | NR | Metachronous  (14) |
| Téglási | 2019 | Hungary | Single-center | 61 | 60 [42-72.5] | 30/31 | 44/5/2 | 0/61/0 | TMA TMA | Brain (61) | Chemotherapy, Radiotherapy, Steroid Treatment | Metachronous  (61) |
| Batur | 2020 | Turkey | Multi-center | 24 | NR | 20/4 | NR | 2/18/4 | 16/8 24/0 | Brain (24) | None | Synchronous |
| Luo | 2020 | China | Single-center | 30(untreated) | 60[34-80] | 82/28 | NR | 7/98/5 | NR NR | Regional lymph nodes (7), distant sites (96), local recurrences (7) | Untreated(30) | Metachronous |
|  |  |  |  | 80(treated) |  |  |  |  |  |  | Targeted drug/chemotherapy (79), Radiotherapy (1) |  |
| Hwang | 2021 | Canada | Multi-center | 27 | NR | NR | NR | 27/0/0 | 27/0 27/0 | NR | Received therapy (9) | NR |
| Shen | 2021 | China | Single-center | 49 | NR | NR | NR | 8/41/0 | 49/0 49/0 | Regional lymph nodes (49) | None | Synchronous |
| Inoue | 2016 | Japan | Multi-center | 132 | NR | NR | NR | NR | TMA TMA | Regional lymph nodes (132) | NR | Synchronous |
| Kim H | 2017 | Korea | Single-center | 37 | 63[33-77] | 27/10 | 26/11/0 | 9/28/0 | 37/0 37/0 | Lung (20), brain (4), pleura (5), and others (8) | NR | Metachronous |

N, number; M, male; F, female; PD-L1, programmed death-ligand 1; SCC, squamous cell carcinoma; ADC, adenocarcinoma; NOS, not otherwise specified; NR, not reported; TMA, tissue microarray; MWA, microwave ablation.

Supplementary Table 2 Pooled discrepancy rates of PD-L1 and direction of discordance from primary lung cancer to paired metastasis in tumor cells

|  | Studies  N | The primary and  paired metastasis  N | Pooled discrepancy rate(95%CI) | I^2^ | P for heterogeneity |
| --- | --- | --- | --- | --- | --- |
| Pooled discrepancy | 14 | 968 | 21%(18-25%) | 54% | <0.05 |
| Positive to negative | 14 | 408 | 26%(22-30%) | 0% | >0.05 |
| Negative to positive | 14 | 560 | 18%(12-24%) | 78% | <0.05 |

Supplementary Table 3 PD-L1 positivity rate between paired primary and metastasis

|  | Study(paired primary tumor and metastasis) | positivity rate(95%CI)-primary lesion | I^2^ | positivity rate(95%CI)-metastatic lesion | I^2^ |
| --- | --- | --- | --- | --- | --- |
| All studies | 15(1029) | 45%(39-52%) | 77% | 43%(37-50%) | 77% |
| ≥50% in tumor cells | 11(710) | 18%(13-24%) | 66% | 19%(15-23%) | 30% |
| ≥1% in tumor cells | 10(741) | 46%(39-55%) | 78% | 44%(37-52%) | 77% |
| ≥5% in tumor cells | 5(288) | 42%(30-59%) | 76% | 41%(28-59%) | 78% |
| E1L3N antibody | 5(524) | 40%(31-52%) | 79% | 39%(30-51%) | 83% |
| 22C3 antibody | 5(286) | 54%(46-65%) | 61% | 53%(44-63%) | 57% |
| SP142 antibody | 3(119) | 32%(23-45%) | 38% | 31%(24-41%) | 0% |
| Metastasis was lymph node | 5(358) | 48%(40-58%) | 61% | 47%(40-56%) | 48% |
| Metastasis was brain | 4(183) | 54%(38-76%) | 84% | 48%(32-71%) | 85% |
